# Forest roadsides harbour less competitive habitats for a relict mountain plant (*Pulsatilla vernalis*) in lowlands

**DOI:** 10.1038/srep31913

**Published:** 2016-08-18

**Authors:** Katarzyna M. Zielińska, Marcin Kiedrzyński, Andrzej Grzyl, Agnieszka Rewicz

**Affiliations:** 1 Department of Geobotany and Plant Ecology, Faculty of Biology and Environmental Protection, University of Lodz, Banacha Str. 12/16, 90-237 Lodz, Poland

## Abstract

The long-term survival of relict populations depends on the accessibility of appropriate sites (microrefugia). In recent times, due to the mass extinction of rare species that has resulted from the loss of natural habitats, the question is – Are there any human-made sites that can act as refugial habitats? We examined forest roadside populations of the mountain plant *Pulsatilla vernalis* in the last large lowland refugium in Central Europe. We compared the habitat conditions and community structure of roadsides with *P. vernalis* against the forest interior. Light availability and bryophyte composition were the main factors that distinguished roadsides. *Pulsatilla* occurred on sites that had more light than the forest interior, but were also more or less shaded by trees, so more light came as one-side illumination from the road. Roadsides had also a lower coverage of bryophytes that formed large, dense carpets. At the same time, they were characterised by a greater richness of vascular plants and ‘small’ bryophytes, which corresponds to a higher frequency of disturbances. In a warming and more fertile Anthropocene world, competition plays the main role in the transformation of forest communities, which is why relict populations have found refugia in extensively disturbed human-made habitats.

Understanding the response of a species to environmental changes is an urgent scientific issue in the context of the comprehensive human impact on ecosystems in the Anthropocene epoch^1^. Natural laboratories for such studies are relict populations – a group of individuals living in isolation after a shift in the species range^2^. Relict populations, and their habitats, harbour the unique story of long-term species persistence when the surrounding conditions become unfavourable; hence, understanding the traits of habitats that allow the long-term existence of isolated populations is of special importance^3^,^4^.

The ability of plants to survive during an unfavourable climatic period is connected with a relatively wide ecological niche^5^ or the high adaptation capabilities of a species^6^, but most of all it is connected with the accessibility of proper sites, i.e. the existence of appropriate microrefugia^7^. In the context of the decline of many natural habitats, the questions are: Are there any elements in the human-structured landscape that could play the role of refugial habitats? If so, which habitat traits are crucial for the maintenance of old, relict populations?

Many studies have confirmed that linear anthropogenic structures such as roadsides, railway embankments or electricity lines may function as secondary habitats for some rare species^8^,^9^,^10^,^11^ or could even have evolutionary importance in local adaptation^12^. Roads that cut across forest complexes constitute more open habitats that can be occupied by some rare species. The impact of such forest roads is not limited to the narrow strip of the road surface, each road creates a road-effect zone that includes the adjacent forest patches that lie alongside the road^13^. Relatively large areas of quite-open and medium-disturbed habitats are created in such a way. They are often characterised by a distinct microclimate and soil conditions in comparison to the forest interior^14^,^15^,^16^. Because roads and their surroundings can be considered to be specific ecosystems with a permanent structure in the forest landscape^14^, we assumed that they could play a refugial role for some relict populations.

In light of the long-term perspective of the survival of a relict population, anthropogenic sites can serve as microrefugia in the landscape if they have conditions that are similar to natural refugial habitats. Plants that are found in anthropogenic sites are commonly the species that ‘use’ such new sites as one of several available habitats in the landscape. However, we can also find species that were previously present on larger areas but are now restricted to such marginal anthropogenic sites. In the second group, there are the light-demanding species of early-successional habitats, whose abundance in the forest interior can be strongly limited by the canopy closure after tree-stand regeneration^16^.

The patterns mentioned above occur in the central European lowlands, where the current types of potential vegetation are closed-canopy temperate forests, which were established during the last millennia, at least since the mid-Holocene period. In those conditions, remnants of open Pleistocene or early-Holocene vegetation (e.g. light-demanding plants) survived mostly in natural tree-less habitats such as sea shores, river gravels, cliffs, bogs, mires and fens^17^,^18^,^19^,^20^,^21^. However, an open character of vegetation was also maintained by the pressure of large herbivores and human activities^22^,^23^. Therefore, traditionally managed forests could harbour the survivors of ancient vegetation^24^,^25^. The character of forest management has changed significantly during the last few decades. The old, traditional practices such as pasturing, litter lacking, coppicing or occasional fires have been abandoned. These were replaced by more mechanistic and systematic forestry, which became the rule. Many examples of the transformation of open woods to closed-canopy stands have been described^26^. One result of the new practices is that heliophilous species have retreated from forest communities and have become endangered, including relict populations that have survived for centuries in the traditionally managed forests and forest gaps. In this situation, relict and light-demanding species can only survive in such marginal sites like roadsides.

One such example is the spring pasque flower *Pulsatilla vernalis* (L.) Mill., which is a mountain plant whose populations occur in the central European lowlands (Fig. 1a) where the species is referred to as a glacial relict. The taxon occurs in alpine grasslands in the mountains, while in lowland localities, it grows in dry pine forests and heathlands (Fig. 1b,c). The extinction of its lowland localities can be observed throughout Europe (Fig. 1a); therefore, the species is listed in the IUCN Red List of Threatened Species in the LC (Least Concern) category^27^. The last large refugium of the occurrence of *Pulsatilla vernalis* in the central European lowlands is in the Tuchola Forest in northern Poland (Fig. 1a) where more than twenty localities still exist. However, as Grzyl and Ronikier^28^ showed, most of these are located in anthropogenically changed sites. Forest roadsides have become especially welcome for *Pulsatilla vernalis* in the current forest landscape of the Tuchola Forest.

In our study we addressed the following questions: 1) How are the habitat conditions and community structure of the roadsides with *Pulsatilla vernalis* and without this relict species different from the forest interior? 2) Are there any diagnostic species for refugial habitats that occur on roadsides?

We understand the forest roadside to be the entire road-effect zone, which includes the edge of a forest and the forest community that surrounds a road. We analysed the habitat and community conditions using a system of research plots. A system of three plots was established in each locality: one plot included the *Pulsatilla* individuals on the roadside, one was located at the same distance from the road but did not contain the relict species and the third plot was established in the forest interior. We measured the light conditions and assessed the shading by trees, shrubs, the coverage of herbaceous vascular plants, bryophytes, lichens, the cover of bare ground as well as the species richness and community composition (total and separately for vascular plants, bryophytes and lichens). In preliminary analyses the traits of bryophytes appeared to be of special importance, so we divided the bryophyte taxa into groups of larger and smaller ones according to their size.

## Results

### Structure of the communities on the roadsides and forest interior

The roadside communities (with and without *Pulsatilla vernalis*) were more species-rich in all of the analysed taxonomic groups in comparison with the forest interior (Table 1, Fig. 2). Plots with *P. vernalis* did not differ significantly from the other plots on the roadsides neither in terms of the number nor coverage of the different taxonomic groups (Fig. 2). At the same time, they were distinguished from the forest interior by a significantly higher number of bryophytes and the coverage of the “small” bryophytes, while the roadside plots without *P. vernalis* specimens were not (Fig. 2a,f). By contrast, a high cover of “large” bryophytes, and hence, the bryophytes as a whole, characterised the forest interior (Fig. 2d,e).

The vertical structure of a habitat did not significantly differ between the forest interior and the roadside (Fig. 3a,b). In the case of tree-layer cover, we did not find any evidence for the significant difference between the plots with *Pulsatilla vernalis* and the forest interior (Fig. 3a,b). However, a clear differentiation between roadsides and forest interior was observed in the case of the light availability index (Fig. 3c).

### Detecting the most significant factors

Multiple stepwise linear regression was carried out in order to determine which of the analysed features of the roadside habitats are of special importance. From several variables that remained after the rejection of insignificant and dependent ones, the analysis determined that three were significant with p < 0.05 (Table 2). The light availability index and number of vascular plants had a negative relationship with the distance from the road, while the cover of ‘large’ bryophytes had a positive relationship.

DCCA revealed a clear gradient along the first axis – from the forest interior (with a high cover of ‘large’ bryophytes) to the roadside plots on which both the light availability and number of vascular plants were higher (Fig. 4). There was no evident differentiation between the plots with *Pulsatilla vernalis* and the other roadside plots. The plots from the forest interior were located close to each other, which suggests the homogeneity of their floristic composition and structure. Conversely, the roadside plots were dispersed, which suggests a pronounced differentiation in species composition and environmental factors (Fig. 4).

The abundance of *Pulsatilla vernalis* (measured by the number of rosettes in a plot) according to the multiple stepwise linear regression depended on only one factor – the cover of ‘large’ bryophytes showed a significant negative relationship (Table 3).

### The diagnostic species of *P. vernalis* roadside localities

A similar analysis was carried out to determine whether there was a relationship between the distance of plots from the road and the occurrence of particular species. The six species with the highest frequency in the plots (*Calluna vulgaris*, *Deschampsia flexuosa*, *Vaccinium vitis-idaea*, *Dicranum polysetum*, *Hylocomium splendens* and *Pleurozium schreberi*) were taken into account. After the rejection of the insignificant ones, the remaining three species were subjected to the multiple stepwise linear regression procedure. All of them appeared to be significantly related to the distance of a plot from the road – *Calluna vulgaris* negatively; *Dicranum polysetum* and *Pleurosium schreberi* positively (Table 4). We also searched for any correlation between the number of *P. vernalis* rosettes and the occurrence of the most frequent species, but we did not find any. If we take the species GLM response to significant environmental factors into account in the DCCA ordination, the light availability index confirms a relationship between the above-mentioned species most clearly (Fig. 5).

## Discussion

In order to obtain the characteristics of anthropogenic habitats with relict populations of *Pulsatilla vernalis*, the difference between roadsides (road-effect zones) with and without this species and forest interior was analysed. Among the analysed biotic and abiotic factors the light availability appeared to be the main one that distinguished the roadsides from the interior of managed forests. However, the vertical structure of communities did not show any significant differences between the cover of the canopy and understory directly above the *Pulsatilla* rosettes and inside the forest. The significant difference between the roadside and forest interior in terms of light conditions was visible after the analysis of the light index as calculated from direct measurements. *Pulsatilla* most often grew under the expanse of tree-crowns but on sites with significant amounts of light coming as a lateral light from the road corridor. The fact that *Pulsatilla vernalis* occurred in habitats that were more or less shaded by the tree crowns in the study area may be an effect of its moisture requirements. The surrounding forest vegetation can be important for the maintenance of adequate moisture conditions during warm periods, and is especially needed for the effective recruitment of seedlings^24^. This statement is supported by the greater share (65%) of more shaded western, northern and eastern expositions in which *Pulsatilla vernalis* survived on the roadsides in the Tuchola Forest.

For the long-term occurrence of *Pulsatilla vernalis* populations in lowlands the light availability seems to be critical or at least a very important factor as for the high-mountain and arctic plant, which grows above the tree-line^6^. Light conditions directly affect the functioning of such species and/or alter biotic interactions^29^,^30^,^31^,^32^. Currently observed trends in plant populations from tundra^33^ showed a climatically induced shift from a competition for soil nutrients to a competition for a light. In the case of *Pulsatilla vernalis*, relict populations have an ability to survive in lowlands after the early-Holocene warming only on poor, sandy soils, where competition was lower and a sufficient amount of light appeared.

**Figure 1:**
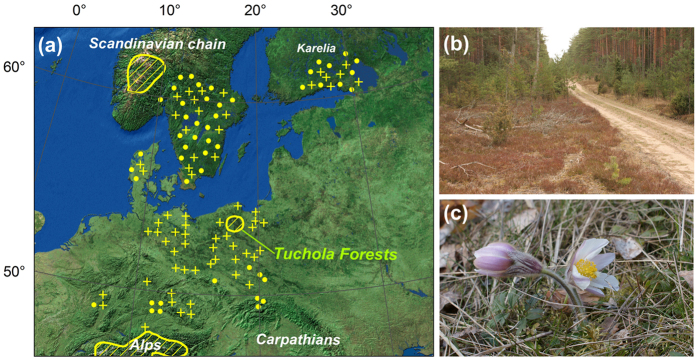
(**a**) The Tuchola Forest – the last large lowland refugium of *Pulsatilla vernalis* in Central Europe. Map generated in ArcMap 9.2 software; map background is the raster layer from Arc Global Data, included in ArcGIS package (ESRI Inc. 1999–2008, Redlands, CA, USA); species distribution layers were created according to Grzyl *et al*.^24^: crosses – extinct localities, dots – isolated localities, solid line – areas with multiple localities; (**b**) Roadside in *Pinus sylvestris* forests – the typical current habitats of *P. vernalis* in the Tuchola Forest; photo M. Kiedrzyński. (**c**) a flowering specimen of *P. vernalis*; photo K.M. Zielińska.

**Figure 2:**
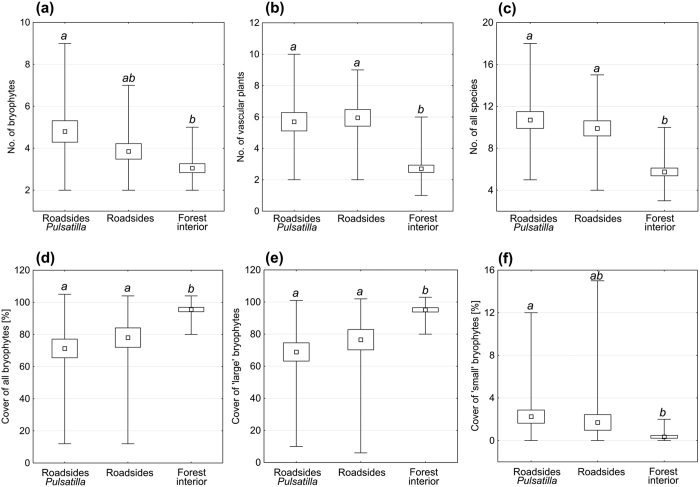
General characteristics (**a–f**) of the habitats on the roadsides with *Pulsatilla vernalis*, roadsides without the taxon and in the forest interior in the Tuchola Forest. Small square – mean value (from twenty 4 m^2^ plots), box – standard error, branches – minimum and maximum values. Different italic letters represent significant (p < 0.05) differences among the types of plots according to the post-hoc tests (multiple comparisons of mean ranks for all groups) in the Kruskal-Wallis rank ANOVA.

**Figure 3:**
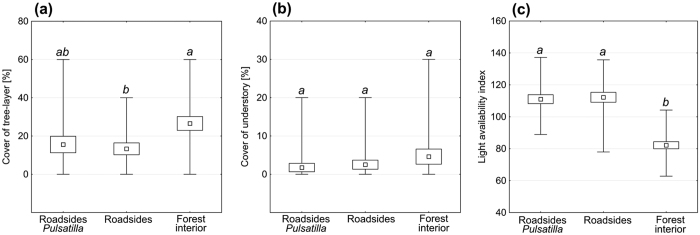
Vertical structure of the habitats (**a,b**) and the light availability index (**c**) on the roadsides with *Pulsatilla vernalis*, roadsides without the taxon and the forest interior in the Tuchola Forest. Small square – mean value (from twenty 4 m^2^ plots), box – standard error, branches – minimum and maximum values. Different italic letters represent significant (p < 0.05) differences among the types of plots according to the post-hoc tests (multiple comparisons of mean ranks for all groups) in the Kruskal-Wallis rank ANOVA.

**Figure 4:**
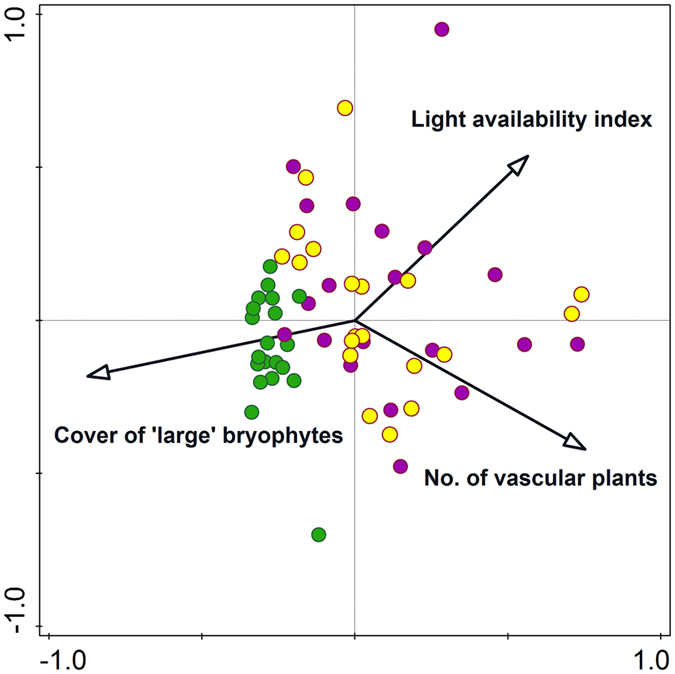
DCCA of the plant composition of the plots and three environmental factors that were significant according to multiple stepwise linear regression (p < 0.05, Table 2) in the relict localities of *Pulsatilla vernalis* in the Tuchola Forest. The types of plots were as follows: roadsides with *Pulsatilla vernalis* (violet circle), roadsides (yellow circle) and the forest interior (green circle).

**Figure 5:**
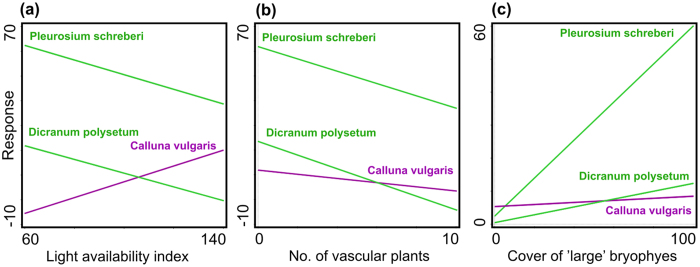
Response curves on the general linear models (GLMs) for species and environmental factors (**a**–**c**) significant according to stepwise linear regression (p < 0.05, Tables 2 and 3) in relict localities of *Pulsatilla vernalis* in the Tuchola Forest.

Light conditions appeared to be important together with a specific structure of the communities, which permitted the functioning of relict populations. In the case of the lowland populations of *Pulsatilla vernalis*, the coverage of bryophytes was of special importance. In lowlands the taxon occurred mostly in *Pinus sylvestris* forest complexes where bryophytes often play a dominant role in the underground^34^. The moss-layer in such communities is mainly formed by dense carpets of highly competitive bryophytes, e.g. *Pleurozium schreberi*, *Hylocomium splendens* or species from the genus *Dicranum*. However, our research showed that the bryophyte coverage was significantly lower in the roadside zones of such forests. This was particularly true in the case of ‘large size’ mosses. On the roadsides we found a significantly lower abundance of *Dicranum polysetum*, which forms cushions consisting of many closely packed vertical stems and *Pleurosium schreberi*, which forms dense carpets of many intertwining, richly branched stems. The influence of a dense moss-layer on *Pulsatilla vernalis* individuals might be two-fold. Firstly, the dense cover of ‘large’ mosses make the successful germination of seeds, which require a barren, extensively disturbed ground impossible^35^,^36^,^37^. Secondly, we observed that growth under the competition of mosses resulted in a thinning and elongating of the main shoots, and also decreased the number of leaves, which had to form long and thin tails that were propped on the moss carpets (Fig. 1c).

It should be pointed out that the areas around roads were characterised by a lower coverage of bryophytes but they had a higher species-richness. The lower abundance of ‘large’ mosses may be of great importance for the existence of other species. The roadsides stood out due to the large number of ‘small’ bryophytes, for example, *Ceraodon purpureus*, *Pohlia nutans* and *Polytichum piliferum*. Such mosses have limited competitive skills and prefer disturbed habitats in lowland landscapes. On the roadsides, we also noted a larger number of vascular plants. The existence of a competitive dependence between vascular plants and bryophytes was described e.g. by Smith *et al*.^38^ or Ingerpuu *et al*.^39^. In our case, the roadsides promoted vascular plants along with a simultaneous decrease of the cover of ‘large’ bryophytes.

One known feature of roadside vegetation is the great richness of species, most of which occur in a random manner (with low frequencies). This pattern was observed by numerous authors^40^,^41^,^42^. The statement is also supported by our DCCA analysis, in which the plots from the roadsides were much more dispersed on the ordination diagram. Therefore, specific species cannot generally be treated as the indicators of “roadside habitats”, but in a comparison to a forest interior, the higher species richness could be a diagnostic feature^40^,^42^,^43^. From among the species that were noted with a higher frequency, only one, *Calluna vulgaris*, was significantly positively related to roadsides. This is not surprising because *Calluna vulgaris* is a spontaneously emerging species in almost all of the larger gaps in the forest canopy of dry pine forests in Central Europe as well as on the edges of forest roads (Fig. 1b).

In a more general view, the community structure of roadside patches with *Pulsatilla* did not differ significantly from the other plots that were located at the roadsides. However, plots with *Pulsatilla vernalis* were characterised by a slightly smaller cover of bryophytes and a slightly higher species number, which suggested a higher degree of disturbances. More detailed analyses, which took into account the division of bryophytes according to their size, showed the strong statistical importance of the lower coverage of ‘large’ bryophytes. Our study proved that in an analysis of the suitability of a habitat to be a microrefugium, an examination of its abiotic features and community composition is insufficient. The particular species traits that are linked to their competitive strength must also be considered. The importance of taking into account the way in which mosses cover the ground (a dense and deep layer or a shallow layer) for their interactions with vascular plants was also found by Gornall *et al*.^44^.

The analysed roadsides can be described as habitats that are characterised by a greater access to light, a higher species diversity and a smaller coverage of bryophytes, which form compact, dense carpets. Such taxonomic and life form diversity is a well-known phenomenon in coniferous forest habitats that have been subjected to a higher frequency of various disturbances^45^. Roadsides in comparison to forest interior are more exposed to small scale disturbances such as the accidental damage of the underground that is caused by road vehicles, the fallout of fine particulates of dust from the road or even occasional small fires. These less competitive habitats, in which patches of barren ground occur, are suitable for the germination of *Pulsatilla vernalis*. These patterns are also supported by the results of Laitinen’s^35^ experiment, in which an ash admixture that was applied to forest soil that was devoid of moss cover and humus significantly enhanced the germination and survival rates of *P. vernalis* seedlings. In lowland localities *Pulsatilla vernalis* was noted on the podzolic soils that can be characterised by a small content of humus, acid or a strongly acid reaction throughout the profile, dryness with moderate porosity and a small capillary water capacity^46^. These stressful conditions prevent *P. vernalis* from strong vascular plants competition. In turn, a reduction of the competition of ‘large’ mosses requires the occurrence of extensive disturbances.

Nowodays roadsides probably became the last suitable habitats for *Pulsatilla vernalis* in the Tuchola Forest. During the periglacial conditions on the foreland of the glacier, *Pulsatilla vernalis* could occur in the cold grasslands and tundra. After that, during the progressive warming and development of forest communities, the light conditions and competition became limiting factors. *Pulsatilla vernalis* survived on poor soils, where the canopy was more open and the competition was lower. Throughout the subsequent centuries, traditional human activity maintained such suitable habitats. However, recent decades, which have seen the application of more schematic forestry, caused the disappearance of the majority of the populations from the forest interior. Our results are consistent with the growing body of evidence that the lowland occurrence of *Pulsatilla vernalis* is connected with disturbances in forests. The maintenance of a species is favoured by periodic fires^37^ or litter removal^36^. In modern forestry occasional fires are efficiently extinguished and litter removal is forbidden. It can be assumed that the loss of appropriate sites is one of, or even the main, reason for the disappearance of *Pulsatilla vernalis*. The question arises of whether the entire network of forest roadsides can be considered to be sufficient refugial habitats, a kind of ‘safe haven’ for the taxon.

Along with protection from the long-term effects of disturbances in current forestry practices^47^ as well as progressive fertilisation^1^, the lowland habitats that are suitable for *Pulsatilla vernalis* are disappearing. The specimens noted during our research probably occurred due to the longevity of the genets. The lack of the recruitment, which was visible by the lack of young specimens, and the low number of flowering individuals as well as the low total number of rosettes, clearly showed that the populations on the roadsides of the Tuchola Forest are also becoming extinct. One reason might be that several crucial factors must occur simultaneously for the long-term maintenance of the species. Despite the suitable habitats, the persistence of *Pulsatilla vernalis* in relict lowland localities depends on the cycles of recruitment that are induced by favourable climatic episodes^24^. Moreover, during such favourable climatic episode, these suitable habitats have to occur in the direct neighborhood of the parent plants, due to the limited ability of seed dispersion.

If the declining trend in the occurrence of the species persists for the next several years, the last large lowland refugium of *Pulsatilla vernalis* in Central Europe will disappear. The active protection of this endangered plant in Europe includes a variety of methods such as litter lacking or controlled fires^36^,^37^ and the reinforcement of the population by planting juvenile individuals that are obtained from local seeds^36^. In the context of climatic changes, we cannot rely only on natural recruitment that is induced by favourable climatic episodes^24^, so planting young specimens that are bred from local seeds together with maintaining of the favourable habitats is necessary. Because of the threat of direct destruction of the plants during the management of roads, surrogate populations should be established in the forest interior using seeds from the roadside populations – in that way the roadside localities became some kind of an important stage in maintaining of this species in lowlands. The identification of habitats outside the roadsides which are suitable for *Pulsatilla vernalis* will be possible when using diagnostic features of the proper sites determined in our study. It is obvious that maybe this will require human assistance in the shaping of such suitable habitats by, for example, thinning of the forest stand.

## Methods

### Studied species and the study area

The spring pasque flower *Pulsatilla vernalis* (L.) Mill. (Ranununculaceae), which is an endemic for Europe, mountain plant, occurs mostly in the subalpine and alpine habitats of the Pyrenees, the Alps, the Carpathians and the Balkans, as well as in the Scandinavian chain. Isolated lowland populations have been observed in Poland, Germany, Norway, Denmark, Sweden and the Russian Karelia^24^,^48^,^49^,^50^,^51^. Nowadays, the species has almost disappeared from the lowland localities in Germany and are found in only a few localities in Denmark. The majority of lowland localities in Poland are presumably extinct, and in the last decade the existence of only 26 populations from the more than 200 that were historically noted has been confirmed. More than 60% of the remaining populations have less than ten individuals and only two have more than 100 rosettes^28^.

The Tuchola Forest, or the Tuchola Pinewoods (in Polish: Bory Tucholskie), which is the last large refugium of the species in the central European Lowlands, is one of the largest forest complexes in northern Poland (an area of more than 120 000 ha). The terrain morphology of the study area was formed during the last glacial period and in the periglacial conditions that prevailed on the foreland of the glacier of the Pomeranian Phase of the Vistulian (Weichselian) glaciations, which occurred ca. 16 ka BP^52^. Nowadays, most of this plain area is covered by fluvioglacial sands. The landscape is also rich in a variety of sand dunes and lakes. The main types of natural potential vegetation are sub-Atlantic pine forests *Leucobryo-Pinetum* and sub-continental oak-pine forests *Querco-Pinetum*^53^.

For many centuries the area has been managed by humans; however, due to the dominance of poor soils in the region, the development of agricultural activity was restricted^54^. Hence, for centuries the human activity connected with the exploitation of the timber was the main pressure in the landscape. In some parts of the Tuchola Forest, this excessive exploitation during the 17^th^ and 19^th^ centuries along with fires caused the deforestation of large areas. Since the beginning of the 20^th^ century, forestry management has become more mechanistic and has included the planned reforestation of open sites^54^. A more or less regular grid of roads was also built and maintained in the Tuchola Forest. These are important for forest managers and for fire protection.

As was mentioned above, the Tuchola Forest is the last large refugium of *Pulsatilla vernalis* in the central European Lowland. The currently known localities of *Pulsatilla vernalis* occur mainly in the central and the southern part of the study area^28^. Dispersion of the populations indicates the ancient high abundance of the species in the region. This statement is confirmed by personal information from the local inhabitants, who state that a few decades ago plentiful occurrences of pasque flowers were observed.

### Sample collection

During the study of the *Pulsatilla vernalis* localities in the Tuchola Forest in 2015, a very small number of individuals was found in the populations. Most of them were growing directly within the roadsides or in the overexposed areas of forest communities in the vicinity of roads or road-like structures such as the deforested strips along railway embankments^28^. Rosettes occurred within 0.5 to 10 m from the road surface. Roads with localities of *Pulsatilla* in their surroundings were of minor transportation value. The majority were the dirt roads used by forest services, small dirt roads along railway tracks and local asphalt roads, the largest of which was 6 m wide. A system of three plots was established – one plot covered a *Pulsatilla* site, one was located the same distance from the road but did not contain the relict species and the third plot was established in the forest interior. The scheme of arrangement of research plots we attached as Appendix 1. The size of each plot was 4 m^2^. The typical distance between the plots was 15 meters but it ranged from 13 to 30 meters. The reason for this was that we did not want to locate research plots in places which were not representative for the whole forest complex, for example on roadsides we passed places recently destroyed by cars. A total of 20 series of three plots were established (60 research plots in total).

The chosen habitat features were assessed in each plot. The general structure of the communities was assessed by estimating the percentage of cover of habitat layers (mosses and lichens, herb-layer, understory, tree-layer). Moreover, the cover of barren, mineral soil was also estimated. The type of soil in those rather poor, sandy sites was similar between the forest edges and interior so the assumption was made that the main abiotic factor differentiating particular plots is the light. The light conditions were measured using a standard digital illuminance meter (STANDARD INSTRUMENTS CO. LTD; AB-1308). Two independent series of light measurements at the distance of 1.5 meters from the ground were taken in a short time in cloudy conditions in each plot. The light index for each plot was calculated as the ratio of the mean value that was counted for the plot to the mean value for the locality (counted as the mean of the values obtained for forest interior plot and one from roadside plots).

The species composition of the underground in each plot was surveyed by listing vascular plants, bryophytes and lichens along with their cover. The cover was estimated within the following percentage scale: 1, 5, 10, 20 …100. In plots with *Pulsatilla vernalis*, the number of rosettes was written down. The complete list of species with their occurrence in the different type of plots is attached in Appendix 2.

### Data analysis

Explanatory variables for determining the differences in habitat conditions between the roadside and forest interior were the light conditions, cover of the tree-layer, undergrowth, coverage of vascular plants, bryophytes, lichens, uncovered mineral soil and species richness (total and separately of vascular plants, bryophytes and lichens).

Because bryophytes were referred to as an important taxonomic group that has an influence on the germination of *Pulsatilla vernalis* in lowlands^35^, a series of analyses of particular bryophyte traits were conducted. In the further analysis, bryophytes were divided into two groups with different competition potentials. The division was determined according to the trait called ‘Length’ in the BRYOATT data basis^55^. ‘Length’ is an indication of size, and is defined as the height of the leafy shoot in acrocarpous mosses or the length of the shoot or thallus in pleurocarpous mosses and liverworts. Bryophytes that were smaller or equal 0.1 m were called ‘small’ and species that were more than 0.1 m were called ‘large’. The assignment of the particular species to the above groups is attached in Appendix 2. In that way, the coverage and number of ‘small’ and ‘large’ bryophytes was added to the above-listed explanatory variables.

Analysis of the differences between the habitat characteristics of plots with *Pulsatilla vernalis* and the two types of correspondence plots was conducted using the nonparametric ANOVA Kruskal-Wallis rank and post-hoc tests. The choice of method was preceded by a conservative Shapiro-Wilk test to assess the compliance of the data distribution with normal distribution. Statistical analyses were performed using the Statistica 10 package^56^.

To determine the importance of the explanatory variables in the differentiation of habitat conditions on the roadsides and in the forest interior, the distance from the research plot to the road was treated as a dependent variable (it ranged from 0.5 to 39 m) in a multiple stepwise linear regression. A similar analysis was carried out for the number of *P. vernalis* rosettes. We also determined whether there was any relationship between the distance from the road (or the number of *P. vernalis* rosettes) and the occurrence of species with a high frequency (occurring in more than 50% of the plots of a given type). The multiple stepwise linear regression was conducted using the vegan package for R 3.0.1 Statistical Software^57^, which was applied after the removal of insignificant (analysis of scatter plots) or correlated (according to VIF) ones from the whole set of variables.

In order to reveal the environmental gradients that determine the variation in the habitats, a constrained multidimensional ordination was performed. The analysis included species composition data and three significant environmental factors according to the multiple stepwise linear regression (p < 0.05, Table 2). Because the CCA resulted in a visible ‘arch effect’, DCCA detrending by the 2^nd^ polynominal was run. Ordinations and species response curves according to environmental factors in the general linear model (GLM) were performed using the CANOCO 5 package^58^.

## Additional Information

**How to cite this article**: Zielińska, K. M. *et al*. Forest roadsides harbour less competitive habitats for a relict mountain plant (*Pulsatilla vernalis*) in lowlands. *Sci. Rep.* **6**, 31913; doi: 10.1038/srep31913 (2016).

## Supplementary Material

Supplementary Information

## Figures and Tables

**Table 1:** Total number of vascular plants, bryophytes and over ground lichens that were noted in particular types of research plots in the Tuchola Forest.

Group of plant	Roadsides with *P. vernalis*	Roadsides	Forest interior	All plots
Vascular plants	33	36	9	43
Bryophytes	16	14	10	19
Lichens	4	2	0	5
*Total*	*53*	*52*	*19*	*67*
Number of plots (each 4 m^2^)	20	20	20	60

**Table 2:**
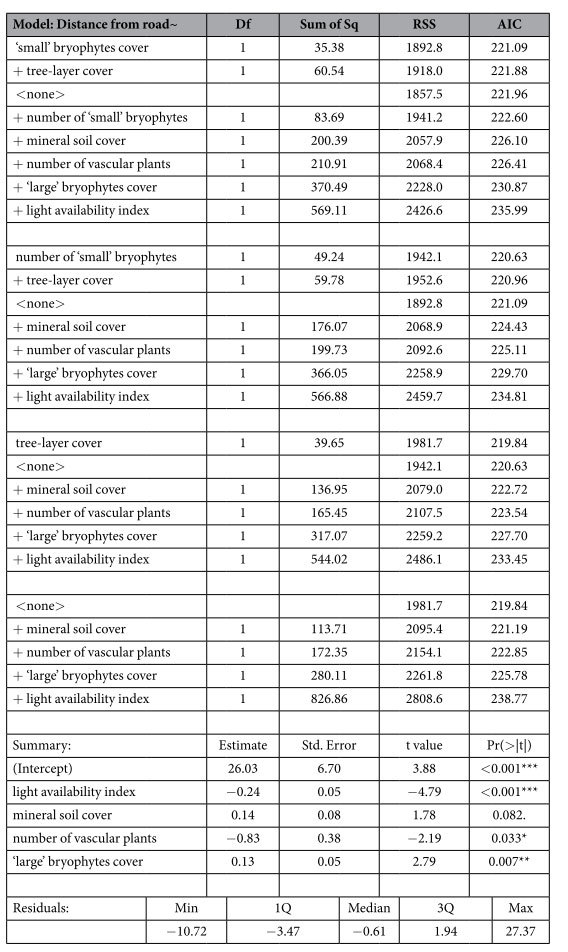
Variables that were significantly dependent on the distance of particular plots from the road surface as obtained by multiple stepwise linear regression.

Residual standard error: 6.003 on 55 degrees of freedom, F-statistic: 18.72 on 4 and 55 DF, p-value: 9.186e-10, Significance codes: ‘***’ 0.001 ‘**’ 0.01 ‘*’ 0.05 ‘.’ 0.1.

**Table 3:**
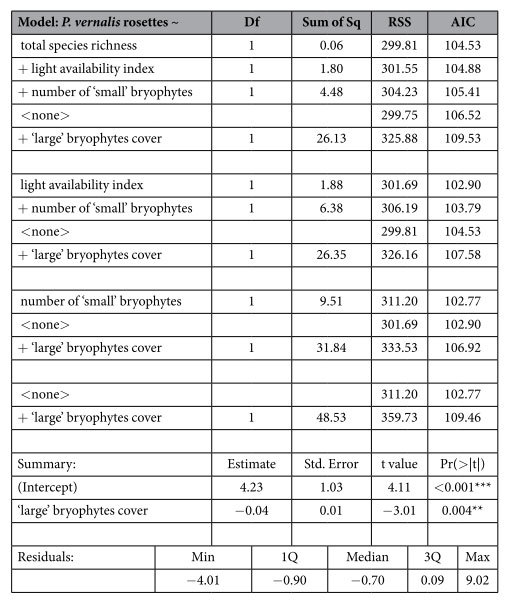
Variables that significantly distinguished the plots with a different number of rosettes as obtained by multiple stepwise linear regression.

Residual standard error: 2.316 on 58 degrees of freedom, F-statistic: 9.044 on 1 and 58 DF, p-value: 0.004, Significance codes: ‘***’ 0.001 ‘**’ 0.01.

**Table 4:**
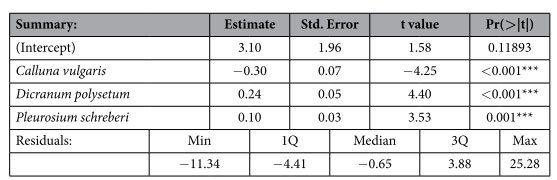
Species that were significantly dependent on the distance of particular plots from the roads as obtained by multiple stepwise linear regression.

Residual standard error: 6.94 on 56 degrees of freedom, F-statistic: 13.73 on 3 and 56 DF, p-value: 7.971e-07 Significance code: ‘***’ 0.001.
