# Supplementary material for: Forest roadsides harbour less competitive habitats for a relict mountain plant (Pulsatilla vernalis) in lowlands
Source: Sci Rep. 2016 Aug 18;6:31913. doi: 10.1038/srep31913 (PMC4989138; doi:10.1038/srep31913)
Supplement: Supplementary Information [file srep31913-s1.doc]

**Forest roadsides harbour less competitive habitats for a relict mountain plant (*Pulsatilla vernalis*) in lowlands**

Katarzyna M. Zielińska, Marcin Kiedrzyński, Andrzej Grzyl, Agnieszka Rewicz

Appendix 1


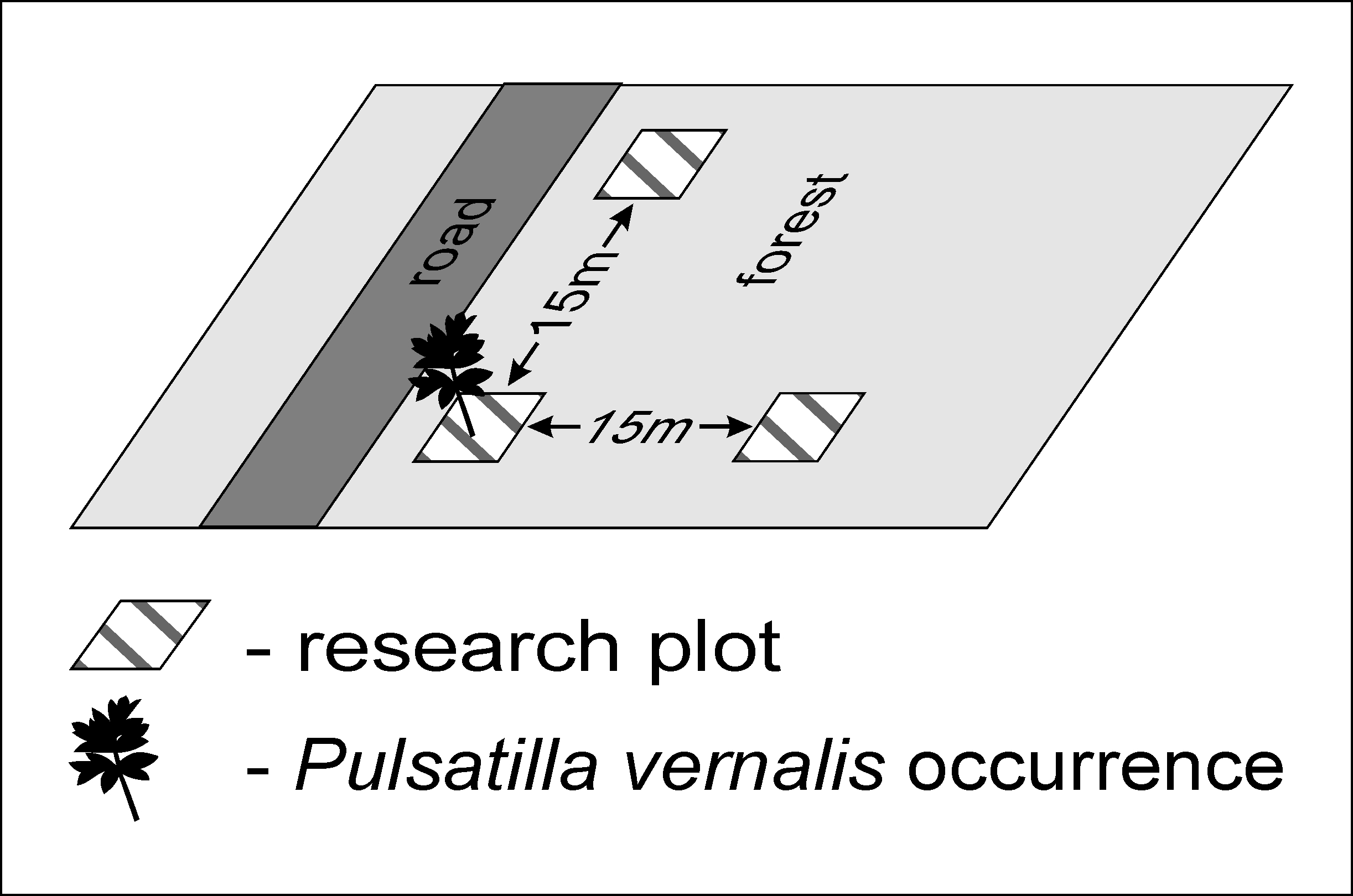


The scheme of three plots location, one with *Pulsatilla vernalis* rosettes, one at the same distance from the road but without *Pulsatilla* specimens and one in the forest interior (out of possible influence of road).The assumed distance from the plot containing the species and the other ones was 15 meters, but we changed it when the principle of representativeness demanded it. For infringement of representativeness of a plot to the forest phytocoenoses or roadside zone we understood any disturbance such as fallen tree etc.

Appendix 2

List of species with their frequency in the different types of research plots. Their affiliation to the group of size ‘large’ or ‘small’ is included after the names of the particular bryophytes. The size assignment was done according to the trait called ‘Length’ in the BRYOATT database (Hill, M. O., Preston, C. D., Bosanquet, S. D. S. & Roy, D. B. *BRYOATT. Attributes of British and Irish mosses, liverworts and hornworts*. Huntingdon: Centre for Ecology and Hydrology, 2007). Bryophytes smaller or equal to 0.1 m (height of the leafy shoot in acrocarpous mosses or the length of the shoot or thallus in pleurocarpous mosses and liverworts) were termed ‘small’ and species above 0.1 m in size were termed ‘large’. The species nomenclature for vascular plants followed the digital version of the Flora Europaea (Tutin TG et al., editors. *Flora Europaea*. Cambridge, http://rbg-web2.rbge.org.uk/FE/fe.html; Accessed: 24th March 2016), for bryophytes followed BRYOATT data basis (Hill et al. 2007), and for the lichens it followed Fałtynowicz (*The lichens, lichenicolous and allied fungi of Poland – an annotated checklist.* W. Szafer Institute of Botany, Polish Academy of Sciences, Kraków, 2003).

| **Group of plant** | **Roadsides with**  ***P. vernalis*** | **Roadsides without**  **P. vernalis** | **Forest interior** |
| --- | --- | --- | --- |
| **Vascular plants** |  |  |  |
| ***Pulsatilla vernalis*** | 20 | 0 | 0 |
| *Achillea millefolium* | 0 | 1 | 0 |
| *Anthoxanthum odoratum* | 1 | 1 | 0 |
| *Arctostaphyllos uva-ursi* | 0 | 2 | 0 |
| *Artemisia absinthium* | 1 | 0 | 0 |
| *Betula pendula* | 1 | 2 | 0 |
| *Calamagrostis arundinacea* | 3 | 4 | 1 |
| *Calamagrostis epigejos* | 0 | 1 | 0 |
| *Calluna vulgaris* | 15 | 16 | 4 |
| *Capsella bursa-pastoris* | 2 | 2 | 0 |
| *Carex pilulifera* | 2 | 1 | 0 |
| *Carex ericetorum* | 4 | 1 | 0 |
| *Cornus sp.* | 0 | 2 | 0 |
| *Dactylis glomerata* | 3 | 2 | 0 |
| *Danthonia decumbens* | 2 | 1 | 0 |
| *Deschampsia flexuosa* | 13 | 11 | 15 |
| *Equisetum hyemale* | 2 | 3 | 0 |
| *Festuca trachyphylla* | 7 | 7 | 0 |
| *Festuca ovina* | 7 | 9 | 2 |
| *Fragaria vesca* | 7 | 6 | 0 |
| *Galium sp.* | 1 | 1 | 0 |
| *Geranium robertianum* | 1 | 0 | 0 |
| *Hieracium pilosella* | 8 | 3 | 0 |
| *Juniperus communis* | 0 | 4 | 1 |
| *Luzula campestris* | 0 | 2 | 0 |
| *Luzula pilosa* | 1 | 1 | 1 |
| *Orthilia secunda* | 1 | 5 | 0 |
| *Pinus sylvestris* | 3 | 3 | 1 |
| *Poa sp.* | 1 | 1 | 0 |
| *Potentilla sp.* | 1 | 0 | 0 |
| *Pteridium aquilinum* | 0 | 1 | 0 |
| *Pulsatilla patens* | 1 | 0 | 0 |
| *Rubus sp.* | 0 | 1 | 0 |
| *Sedum acre* | 1 | 0 | 0 |
| *Stellaria media* | 2 | 2 | 0 |
| *Solidago virgaurea* | 1 | 0 | 0 |
| *Tanacetum vulgare* | 0 | 1 | 0 |
| *Thymus serpyllum* | 1 | 1 | 0 |
| *Trifolium repens* | 0 | 1 | 0 |
| *Vaccinium myrtillus* | 2 | 2 | 9 |
| *Vaccinium vitis-idaea* | 15 | 16 | 20 |
| *Veronica officinalis* | 1 | 1 | 0 |
| *Vicia sp.* | 2 | 1 | 0 |
| *Viola canina* | 1 | 0 | 0 |
| **Bryophytes** |  |  |  |
| *Aulacomium palustre* – small | 1 | 1 | 0 |
| *Brachythecium albicans* – small | 6 | 4 | 0 |
| *Brachythecium rutabulum* – large | 1 | 2 | 0 |
| *Brachythecium salebrosum* – small | 0 | 1 | 0 |
| *Brachythecium starkei* subsp. *oedipodium* – small | 8 | 5 | 3 |
| *Ceratodon purpureus* – small | 2 | 0 | 0 |
| *Dicranum polysetum* – large | 18 | 17 | 18 |
| *Hylocomium splendens* – large | 10 | 12 | 10 |
| *Hypnum jutlandicum* – small | 3 | 0 | 3 |
| *Leucobryum glaucum* – large | 0 | 0 | 1 |
| *Plagiomnium affine* – small | 7 | 5 | 0 |
| *Pleurozium schreberi* – large | 20 | 19 | 20 |
| *Pohlia nutans* – small | 2 | 0 | 0 |
| *Polytrichum formosum* – small | 0 | 1 | 1 |
| *Polytrichum juniperinum* – small | 3 | 0 | 0 |
| *Polytrichum piliferum* – small | 1 | 1 | 0 |
| *Pseudoscleropodium purum* – large | 6 | 4 | 2 |
| *Rhytidiadelphus squarrosus* – large | 2 | 1 | 2 |
| *Ptilidium ciliare* (liverwort) – small | 6 | 4 | 1 |
| **Lichens** |  |  |  |
| *Cladonia arbuscula* | 1 | 0 | 0 |
| *Cladonia digitata* | 1 | 0 | 0 |
| *Cladonia furcata* | 1 | 0 | 0 |
| *Cladonia rangiferina* | 1 | 1 | 0 |
| *Cladonia squamosa* | 0 | 1 | 0 |
| **Vascular plant richness** | 33 | 36 | 9 |
| **Bryophytes richness** | 16 | 14 | 10 |
| **Lichens richness** | 4 | 2 | 0 |
| **Total species richness** | 53 | 52 | 19 |
